# Mobile Health Tool to Capture Social Determinants of Health and Their Impact on HIV Treatment Outcomes Among People Who Use Drugs: Pilot Feasibility Study

**DOI:** 10.2196/59953

**Published:** 2025-03-26

**Authors:** Rachel E Gicquelais, Caitlin Conway, Olivia Vjorn, Andrew Genz, Gregory Kirk, Ryan Westergaard

**Affiliations:** 1 School of Medicine and Public Health, University of Wisconsin-Madison, 603 WARF Office Building, 610 Walnut Street, Madison, WI, 53726, United States, 1 608-890-1837; 2 University of Wisconsin-Madison School of Nursing, Madison, WI, United States; 3 Center for Health Enhancement Systems Studies, University of Wisconsin-Madison College of Engineering, Madison, WI, United States; 4 Department of Epidemiology, Johns Hopkins Bloomberg School of Public Health, Baltimore, MD, United States; 5 Division of Infectious Diseases, Johns Hopkins University School of Medicine, Baltimore, MD, United States

**Keywords:** HIV, drug use, social determinants of health, mobile health, mHealth, smartphone

## Abstract

**Background:**

Active substance use, food or housing insecurity, and criminal legal system involvement can disrupt HIV care for people living with HIV and opioid use disorder (OUD). These social determinants of health are not routinely captured in clinical settings.

**Objective:**

We evaluated whether real-time reports of social and behavioral factors using a smartphone app could predict viral nonsuppression and missed care visits to inform future mobile health interventions.

**Methods:**

We enrolled 59 participants from the AIDS Linked to the Intravenous Experience (ALIVE) Study in Baltimore, Maryland, into a 12-month substudy between February 2017 and October 2018. Participants were eligible if they had OUD and had either a measured HIV RNA ≥1000 copies/mL or a ≥1-month lapse in antiretroviral therapy in the preceding 2 years. Participants received a smartphone and reported HIV medication adherence, drug use or injection, and several disruptive life events, including not having a place to sleep at night, skipping a meal due to lack of income, being stopped by police, being arrested, or experiencing violence on a weekly basis, through a survey on a mobile health app. We described weekly survey completion and investigated which factors were associated with viral nonsuppression (HIV RNA ≥200 copies/mL) or a missed care visit using logistic regression with generalized estimating equations adjusted for age, gender, smartphone comfort, and drug use.

**Results:**

Participants were predominantly male (36/59, 61%), Black (53/59, 90%), and had a median of 53 years old. At baseline, 16% (6/38) were virally unsuppressed. Participants completed an average of 23.3 (SD 16.3) total surveys and reported missing a dose of antiretroviral therapy, using or injecting drugs, or experiencing any disruptive life events on an average of 13.1 (SD 9.8) weekly surveys over 1 year. Reporting use of any drugs (adjusted odds ratio [aOR] 2.3, 95% CI 1.4‐3.7), injecting drugs (aOR 2.3, 95% CI 1.3‐3.9), and noncompletion of all surveys (aOR 1.6, 95% CI 1.1‐2.2) were associated with missing a scheduled care visit over the subsequent 30 days. Missing ≥2 antiretroviral medication doses within 1 week was associated with HIV viral nonsuppression (aOR 3.7, 95% CI: 1.2‐11.1) in the subsequent 30 days.

**Conclusions:**

Mobile health apps can capture risk factors that predict viral nonsuppression and missed care visits among people living with HIV who have OUD. Using mobile health tools to detect sociobehavioral factors that occur prior to treatment disengagement may facilitate early intervention by health care teams.

## Introduction

In 2019, the US Department of Health and Human Services launched the Ending the HIV Epidemic initiative, establishing a goal to reduce new infections of HIV in the United States by 90% by the year 2030 [1]. Achieving this ambitious goal hinges on rapid linkage of newly diagnosed individuals to antiretroviral therapy (ART) and sustained HIV viral suppression to prevent transmission. Active injection drug use and use of other substances, such as alcohol, are associated with inconsistent adherence to ART and viral nonsuppression [2-5]. Accordingly, people who use drugs and are living with HIV are among the most vulnerable to disruptions in care that threaten sustained viral suppression.

The link between drug and alcohol use and viral nonsuppression often relates to unmet needs broadly defined as social determinants of health [6]. For example, housing instability among people who use drugs has been linked to an increased risk of both acquiring HIV and viral nonsuppression [6]. Injecting drugs raises the likelihood of unstable housing and hinders improvements in housing situations among people living with HIV [7,8], thereby threatening viral suppression. Further, inadequate access to food is highly prevalent among people who use drugs [9,10] and has been associated with viral nonsuppression [11], mortality [12], and engaging in behaviors that raise HIV transmission risk, such as receptive syringe sharing [13] and unprotected sexual intercourse [14]. The criminalization of drug use and social stigma of HIV further contribute to difficulties in engaging people who use drugs in both HIV and substance use disorder treatment [15] as well as to disruptions in care due to arrest or incarceration [16,17]. The link between social determinants of health and patients’ capacity to engage and sustain in HIV care illustrates a need for innovative strategies that provide timely support to address the root causes of care disruption and viral nonsuppression.

Mobile health (mHealth) is defined broadly by the World Health Organization as “mobile and wireless technologies to support the achievement of health objectives” [18]. mHealth tools can provide a platform that facilitates real-time detection and the provision of timely assistance when events that could disrupt care engagement occur. Further, information captured through mHealth apps could detect early warning signs of care disengagement, such as missed ART doses or increases in drug use. These technologies may be particularly well-suited to augment existing patient-centered, comprehensive models of HIV care that are enabled by federal Ryan White funding in the United States [19], as these care models already feature medical case management, food pantries, support to obtain housing, and other services to address social determinants of health. In the present pilot feasibility study, we explored the potential of a smartphone app to detect disruptive life events and early warning signs of HIV care disengagement over a 1-year period among 59 people who use drugs living with HIV. Using a weekly survey within a smartphone app designed to support substance use disorder recovery, we assessed how often people who use drugs used the smartphone app and reported experiencing disruptive life events. Through subsequent linkage with electronic health record data, we assessed whether these disruptive life events were associated with 2 outcomes representing care disengagement: viral nonsuppression and missed care visits.

## Methods

### Participants, Recruitment, and Follow-Up

This manuscript reports on a pilot feasibility study of an mHealth app designed to detect disruptive events that could threaten sustained engagement in HIV care and examine the preliminary associations of these disruptive events with HIV care engagement. Participants for this study came from the AIDS Linked to the IntraVenous Experience (ALIVE) study, a prospective cohort study of people who inject drugs in Baltimore, Maryland, that has been described previously [20]. A total of 5506 individuals have been enrolled since the study began in 1988, and approximately 524 were living and had HIV at the time enrollment for the present substudy, which began in 2017 [21]. To enroll in the ALIVE study, participants must be aged 18 years or older and have a history of injection drug use. Participants are tested for HIV at enrollment. Follow-up is ongoing and involves twice-yearly visits to the ALIVE clinic where participants complete surveys about health history in the past 6 months and repeat HIV testing. They also provide consent for the researchers to access medical records to obtain health care utilization data.

The present analysis includes 59 ALIVE study participants living with HIV who were recruited through the HIV care clinic affiliated with Johns Hopkins Hospital in Baltimore, Maryland, United States. Medical records from ALIVE participants who had received HIV care at Johns Hopkins were reviewed to determine eligibility. If eligible, they were invited to meet with a study coordinator to complete a brief screening survey and enroll in the study. Recruitment flyers were also posted in the ALIVE study office and the Johns Hopkins clinic, inviting patients to self-refer for eligibility screening. The inclusion criteria were (1) ever had opioid use disorder (OUD), (2) used a nonprescribed opioid in the past year, (3) not treated with ART for ≥1 month in the past 2 years or had an HIV RNA viral load of ≥1000 copies/mL in the past 2 years, (4) a patient of the local HIV clinic from which participants were recruited, and (5) a participant of the ALIVE study.

All enrolled substudy participants received a smartphone with an unlimited voice and data plan and underwent a brief training with a study coordinator on using the study mHealth app. Participants completed an interviewer-administered baseline survey and follow-up surveys every 3 months for a 1-year period. The study team received HIV viral load testing results from the electronic health record at the local HIV clinic where recruitment was conducted and from routine semiannual testing performed as part of the ALIVE study follow-up. The date of all scheduled care visits at the local HIV clinic and whether visits were missed or attended was also collected. Enrollment and follow-up occurred during 2017 and 2018.

### Ethical Considerations

This study was approved by the institutional review boards at the University of Wisconsin-Madison (protocol ID: 2016‐1190) and Johns Hopkins University (protocol ID: IRB00007523). Results were reported using the CONSORT (Consolidated Standards of Reporting Trials) guidelines for pilot studies. Written informed consent was obtained from all individual participants included in the study. All data collected were coded using a unique study identification number to ensure that individual participants could not be identified. Participants were compensated US $20 cash for completing a baseline survey and another US $20 for each completed follow-up survey. They also received a bonus of US $10 remuneration for each completed survey at the end of the 1-year follow-up period (up to US $50).

### The A-CHESS mHealth App

The mHealth system used in this study, the Addiction-Comprehensive Health Enhancement Support System (A-CHESS), was designed to support recovery from alcohol use disorder and was found to reduce drinking in a prior study of people with alcohol use disorder [22]. A-CHESS has since been adapted for several other risk groups, including those living with OUD [23,24]. The theoretical framework supporting A-CHESS is the self-determination theory [25], which emphasizes that adaptive functioning to manage health conditions is improved by meeting 3 needs: competence (ie, the ability to cope with challenges), social relatedness (ie, having meaningful relationships and a feeling of connection), and autonomy (ie, a sense of control over behavior and goals). A-CHESS is therefore organized around these 3 pillars. Competence is supported by an extensive resource library containing podcasts, information, and other resources to support recovery. An in-app discussion board and private messaging function support relatedness by allowing app users to connect with one another and with case managers or providers who also have accounts on the app. Weekly self-monitoring surveys and a goal-setting feature within the app provide opportunities to build autonomy. Participants receive notifications to complete the self-monitoring survey on a weekly basis. For this study, the weekly self-monitoring survey was adapted to investigate potentially disruptive events that could threaten sustained engagement in HIV care, such as being arrested or not having a place to live, as well as events that may signal or raise risk of HIV care disengagement, such as missed ART doses and drug use (further described in Measures). Participants were invited to use A-CHESS on their study-provided cell phone for 12 months.

### Measures

At the study enrollment visit, participants provided informed consent and completed a survey administered by study staff. They reported their HIV diagnosis date and whether they were currently using ART. Regarding drug use, participants reported whether they had used any illegal or street drugs (including marijuana) or used any medications not as prescribed in the past 30 days, what drugs they used, and whether they were currently taking any medications for OUD (including methadone, buprenorphine, or naltrexone). Participants also reported whether they had ever been diagnosed with any mental health conditions, including anxiety, depression, bipolar disorder, attention deficit hyperactivity disorder, obsessive-compulsive disorder, panic disorder, or post-traumatic stress disorder. Participants reported their comfort level using a smartphone on a numeric scale (1=not at all comfortable) to (5=very comfortable). Finally, participants reported sociodemographic characteristics, including age, gender, race, ethnicity, education, and employment.

The 2 primary outcomes of the study were abstracted through a review of electronic health records and ALIVE semiannual study visit data available for each participant during the 1-year follow-up period. These included viral nonsuppression, defined by the Centers for Disease Control and Prevention as an HIV viral load of ≥200 copies/mL (vs viral load of <200 copies/mL) [26] and missing a scheduled care visit, defined as missing any scheduled visit to the local HIV care clinic affiliated with the study (vs attending the visit). The HIV viral load at baseline was considered the closest HIV measurement to a participant’s baseline survey within the period of 1 year before to 7 days after study enrollment. Electronic health records were also used to characterize baseline CD4+ count defined as the closest CD4 measurement to a participant’s baseline survey within the 1 year before to 60 days after the baseline date. For the missed visit outcome, a total of 841 care visits were included in the analysis. These 841 visits include scheduled visits (regardless of visit attendance) with a medical provider, nurse, case manager, or mental health provider to reflect the breadth of care that patients living with HIV may receive through the United States Health Resources & Services Administration Ryan White HIV/AIDS Program. The primary outcome was whether a scheduled visit was missed versus attended. A total of 147 scheduled visits that were canceled before the appointment occurred were excluded from analyses.

The weekly self-monitoring survey included questions about the number of days ART was not taken, any drug use (defined by combining separate questions on use of heroin, any other opioids (including oxycodone, morphine, fentanyl, etc), cocaine, methamphetamine, or sedatives (including Valium, Ativan, or Xanax)), any injection drug use, and several potentially disruptive life events over the past 7 days. Potentially disruptive life events included not having a place to sleep, skipping a meal because of not having enough income, being stopped by police, being put in jail, and being robbed or beat up. Responses to questions on the weekly self-monitoring survey were used to form several binary indicator variables summarizing whether events of interest were reported on any weekly survey completed in the 30 days prior to having a viral load measurement and separately, in the 30 days prior to a scheduled care visit. Binary variables for noncompletion of all weekly surveys in the 30 days prior to each outcome and for reporting any significant event (ie, missed ART dose, any drug use, injecting drugs, or any disruptive life event) in the 30 days prior to each outcome were also created. These indicators were analyzed as the primary independent variables for the study. A-CHESS system use was summarized as the average number of days A-CHESS was used and the average number of weekly surveys completed per participant over the entire 1-year study period and per study month.

### Analysis

Descriptive characteristics of the sample at baseline were calculated and compared between those who had at least 1 HIV viral load ≥200 copies/mL (vs those for whom all HIV test results across the 1-year study period indicated viral suppression). These characteristics were also compared for those who missed ≥50% of their care visits (vs <50%). Engagement with the A-CHESS smartphone app was described in the total sample and by the primary outcomes. To characterize whether reporting missed ART doses, any drug use, injecting drugs, or experiencing a disruptive life event were associated with viral nonsuppression or a missed care visit, logistic regression with generalized estimating equations accounting for repeated outcome measurements for each participant were used to estimate odds ratios with 95% CIs, as participants could have multiple HIV viral loads taken or care visits scheduled over the 1-year follow-up period. Unadjusted models and models adjusted for several confounders selected a priori were computed. Confounders included in adjusted models were sociodemographic characteristics (age, race, ethnicity, and gender), comfort using a smartphone (treated as a continuous variable with a range of 1-5), and whether the participant had used any drugs in the past 30 days, all of which were reported at study enrollment. In a sensitivity analysis, models were run with an alternate definition of HIV viral nonsuppression, ≥1000 copies/mL, as this matched one of the eligibility criteria for the study and was used to define virological failure in a prior, foundational study of HIV transmission [27].

## Results

### Participant Characteristics

A total of 59 participants enrolled in the 1-year study (Table 1). At baseline, participants were a mean age of 53 years and predominantly male (n=36, 61%), Black race (n=53, 90%), and non-Hispanic ethnicity (n=54, 92%). Approximately 52% (n=31) had less than a high school education, and only 5% (n=3) were employed at the time of the study. In the month before enrollment, 83% (n=49) had used any drugs, 71% (n=42) used opioids, 48% (n=28) used stimulants, and 27% (n=16) had injected drugs. While nearly all (n=55, 93%) reported currently taking ART, 16% (n=6) were virally nonsuppressed (defined as having a viral load of ≥1000 copies/mL at baseline), and 20% (n=10) had a CD4+ count <200 cells/mm^3^. Participants had been living with HIV for a mean of 19 years prior to study enrollment, with 8% (n=5) having been diagnosed in the last 5 years.

**Table 1.** Descriptive characteristics at baseline among a sample of 59 people living with HIV and opioid use disorder enrolled in a pilot feasibility study of the A-CHESS (Addiction-Comprehensive Health Enhancement Support System) mobile health app during 2017 and 2018.

Characteristic	Total (n=59)	HIV viral suppression		Missed care visits	
		≥1 test ≥200 copies/mL (n=25)	All tests <200 copies/mL (n=27)	Missed ≥50% visits (n=23)	Missed <50% visits (n=33)
Age (years), median (IQR)	53 (11)	53 (9.0)	53 (12.5)	51 (10.0)	53 (11.1)
Female (vs male), n (%)	23 (39)	11 (44)	10 (37)	7 (30)	15 (46)
Black or African American race (vs another race), n (%)	53 (90)	25 (100)	24 (89)	21 (91)	29 (88)
Hispanic or Latinx (vs non-Hispanic), n (%)	5 (8)	0 (0)	4 (15)	2 (9)	3 (9)
Less than high school education (vs high school or more), n (%)	31 (52)	12 (48)	14 (52)	12 (52)	17 (52)
Employed (vs unemployed), n (%)	3 (5)	1 (4)	2 (7)	3 (13)	0 (0)
Comfort using a smartphone, mean (SD)	3.8 (1.3)	3.8 (1.4)	3.7 (1.3)	3.8 (1.3)	3.9 (1.3)
Any mental health concerns (vs none), n (%)	40 (68)	15 (60)	20 (74)	15 (65)	25 (76)
Used drugs in past 30 days (vs none), n (%)	49 (83)	19 (76)	25 (93)	20 (87)	27 (82)
Used opioids in past 30 days (vs none), n (%)	42 (71)	16 (64)	22 (82)	16 (70)	25 (76)
Used stimulants in past 30 days (vs none), n (%)	28 (48)	11 (44)	15 (56)	13 (56)	15 (46)
Injected drugs in past month (vs not), n (%)	16 (27)	6 (24)	7 (26)	7 (30)	8 (24)
Years living with HIV, mean (SD)	19.4 (8.7)	19.1 (7.5)	20.0 (9.2)	17.8 (7.2)	20.4 (9.1)
Currently taking antiretroviral therapy (vs not), n (%)	55 (93)	26 (96)	23 (92)	22 (96)	30 (91)
HIV RNA ≥1,000 copies/mL^a^ (vs<1,000), n (%)	6 (16)	5 (29)	1 (6)	2 (14)	4 (19)
CD4+ count < 200cells/mm^3^^b^ (vs ≤200), n (%)	10 (20)	8 (36)	1 (4)	8 (40)	1 (4)

a 37 participants had an available HIV viral load result at baseline.

b 50 participants had an available CD4 count at baseline.

Of 52 participants who had at least 1 HIV viral load test during follow-up (comprising 237 total viral load tests), nearly half of the participants (n=25, 48%) had at least 1 detectable viral load of ≥200 copies/mL during the follow-up period. Regarding missed care visits, of 56 participants who had a visit scheduled during the follow-up period (comprising 841 total scheduled visits), 41% (n=23) of participants missed at least half of their scheduled care visits during the study period. The modest sample size limited our ability to test for differences in baseline characteristics between those who were virally nonsuppressed at least once over the follow-up period or who missed over half of their care visits, as shown in Table 1.

### A-CHESS Engagement and Weekly Reports of Missed ART Doses, Drug Use, and Disruptive Life Events

Over the 1-year follow-up period, participants used A-CHESS an average of 119.4 (SD 82.8) total days and on an average of 11.5 (SD 4.4) days per month. The weekly survey was completed an average of 23.3 (SD 16.3) times per participant across the follow-up period, leaving a total of 1353 weekly surveys available for analysis (Table 2). In total, 46.3% (627/1353) of weekly surveys included a report of missed ART doses, drug use, or a disruptive life event. Missing a dose of ART was reported on 25.2% (341/1353) of surveys, an average of 8.5 (SD 7.1) times per participant across the study period. Using any drugs was reported an average of 9.5 (SD 9.3) times per participant across the study period, and injecting drugs was reported 5.8 (SD 8.2) times per participant. Skipping a meal was reported an average of 6.6 (SD 6.5) times per participant. Not having a place to sleep was reported an average of 2.7 (SD 3.4) times per participant. Other disruptive life events (ie, being put in jail and being robbed or beaten up) were reported <2 times, on average, per participant.

**Table 2.** Missed antiretroviral therapy (ART) doses, drug use, and disruptive life events reported on weekly surveys completed by 59 participants living with HIV and opioid use disorder enrolled in a pilot feasibility study of the A-CHESS (Addiction-Comprehensive Health Enhancement Support System) mobile health app during 2017 and 2018.

Survey item	Number of weekly surveys, n (%)	Number of weekly surveys completed per participant, mean (SD)
Total number of weekly surveys completed	1353 (100)	23.3 (16.3)
Reported any missed ART, drug use, or disruptive life event	627 (46.3)	13.1 (9.9)
1 or more missed ART doses	341 (25.2)	8.5 (7.1)
2 or more missed ART doses	190 (14)	5.9 (6.1)
Any drug use	417 (30.8)	9.5 (9.3)
Injected drugs	116 (8.6)	5.8 (8.2)
Did not have a place to sleep at night	27 (2)	2.7 (3.4)
Skipped a meal because I didn’t have enough money	131 (9.7)	6.6 (6.5)
Was stopped by police	22 (1.6)	1.8 (1.2)
Was put in jail	9 (0.7)	1.5 (0.6)
Was robbed or beat up	13 (1)	1.6 (0.9)

Table S1 in Multimedia Appendix 1 shows differences in A-CHESS utilization frequency and weekly survey completion during follow-up between participants who were virally nonsuppressed at least once over the follow-up period (vs suppressed throughout follow-up) as well as between those who missed 50% or more of their scheduled care visits (vs who missed fewer than 50% of their care visits). These comparisons indicate that more weekly surveys were completed by those who did not have any viral loads ≥200 copies/mL (*P*=.01) and who did not miss any care visits (*P*=.005). Those who did not miss any care visits during follow-up also tended to use A-CHESS more often (*P*=.03).

### Association of Missed ART, Drug Use, and Disruptive Life Events Reported Through A-CHESS With Missed Care Visits and Viral Nonsuppression

In bivariable and adjusted analyses, injecting drugs, any drug use, and not completing any weekly surveys in the prior month were associated with missing a scheduled care visit in the subsequent 30 days (Table 3). Specifically, injecting drugs was associated with 2.3-fold higher adjusted odds (95% CI 1.3‐3.9) and using any drugs was associated at a similar magnitude with missing a scheduled care visit after adjustment for age, comfort using a smartphone, gender, and any drug use at baseline. Not completing the weekly survey (vs completing at least 1 weekly survey) was associated with 57% higher odds of missing a care visit in the following 30 days (adjusted odds ratio [aOR] 1.6, 95% CI 1.1‐2.2).

**Table 3.** Association of reporting missed antiretroviral therapy (ART) doses, drug use, and disruptive life events in the prior 30 days with missing a care visit among 59 participants living with HIV and opioid use disorder enrolled in a pilot feasibility study of the A-CHESS (Addiction-Comprehensive Health Enhancement Support System) mobile health app during 2017 and 2018.

Events reported in the month prior to scheduled care visit^a^	Unadjusted OR^b^ (95% CI)	Adjusted OR (95% CI)^c^
Any missed ART, drug use, or disruptive life event	1.20 (0.80‐1.81)	1.25 (0.81‐1.93)
1 or more missed ART doses	0.91 (0.61‐1.35)	0.89 (0.57‐1.39)
2 or more missed ART doses	1.22 (0.70‐2.15)	1.30 (0.69‐2.43)
Any drug use	2.07 (1.28‐3.35)	2.27 (1.41‐3.66)
Injected drugs	2.11 (1.24‐3.59)	2.26 (1.30‐3.91)
Did not have a place to sleep at night	1.87 (0.38‐9.27)	1.76 (0.28‐11.14)
Skipped a meal due to not enough money	0.76 (0.36‐1.60)	0.81 (0.37‐1.74)
Stopped by police	1.07 (0.45‐2.54)	1.11 (0.42‐2.97)
No weekly surveys completed in past 30 days	1.42 (1.00‐2.02)	1.57 (1.10‐2.22)

a Odds ratios are reported relative to a referent group that completed at least 1 weekly survey in the 30 days prior to a scheduled care visit but did not indicate experiencing the disruptive life event.

b OR: odds ratio.

c Adjusted for age, gender, comfort using a smartphone (score: 1=not at all comfortable to 5=very comfortable), and any drug use in the past 30 days, all of which were reported at study enrollment.

In both unadjusted and adjusted analyses, missing 2 or more doses of ART was associated with having a nonsuppressed viral load in the subsequent 30 days (aOR 3.7, 95% CI 1.2‐11.1; Table 4). These associations were similar in a sensitivity analysis in which the outcome was defined as a viral load of load ≥1000 (Table S2 in Multimedia Appendix 1). Notably, we were unable to adjust for race or ethnicity given the predominantly Black and non-Hispanic racial or ethnic makeup of the sample (90% Black and 8% Hispanic). Further, because being put in jail and robbed or beaten up were each reported on only a few surveys (<15 surveys each), we were unable to analyze their relationship with the primary outcomes of interest.

**Table 4.** Association of reporting missed antiretroviral therapy (ART) doses, drug use, and disruptive life events in the prior 30 days with HIV viral nonsuppression (viral load ≥200 copies/mL) among 59 participants living with HIV and opioid use disorder enrolled in a pilot feasibility study of the A-CHESS (Addiction-Comprehensive Health Enhancement Support System) mobile health app during 2017 and 2018.

Events reported in the month prior to viral load measurement^a^	Unadjusted OR^b^ (95% CI)	Adjusted OR (95% CI)^c^
Any missed ART, drug use, or disruptive life event	1.30 (0.72‐2.33)	1.28 (0.68‐2.44)
1 or more missed ART doses	1.96 (0.88‐4.35)	1.92 (0.76‐4.76)
2 or more missed ART doses	4.00 (1.61‐10.00)	3.70 (1.23‐11.11)
Any drug use	0.93 (0.46‐1.85)	0.92 (0.44‐1.92)
Injected drugs	0.79 (0.22‐2.86)	0.74 (0.19‐2.78)
Did not have a place to sleep at night	2.38 (0.56‐10.00)	2.44 (0.54‐11.11)
Skipped a meal due to not enough money	0.64 (0.25‐1.67)	0.6 (0.22‐1.64)
Stopped by police	1.72 (0.34‐8.33)	1.67 (0.32‐9.09)
No weekly surveys completed in past 30 days	0.94 (0.53‐1.69)	0.87 (0.46‐1.64)

a Odds ratios are reported relative to a referent group that completed at least 1 weekly survey in the 30 days prior to a viral load test but did not indicate experiencing the disruptive life event.

b OR: odds ratio.

c Adjusted for age, gender, comfort using a smartphone (score: 1=not at all comfortable to 5=very comfortable), and any drug use in the past 30 days, all of which were reported at study enrollment.

## Discussion

This study is a proof-of-concept demonstrating the utility and feasibility of using an mHealth system to capture time-varying threats to HIV viral suppression and care engagement among people living with HIV and OUD. Our system focused on detecting early warning signs of HIV care disengagement and disruptive events that fall within the realm of social determinants of health known to impact the health of people who use drugs [6]. Over a 1-year period, we captured >600 reports of events that could be disruptive to HIV care and be early warning signs of care disengagement. Using or injecting drugs and reporting missed doses of ART were associated with an elevated likelihood to miss a care visit or be virally nonsuppressed during follow-up, respectively. Not completing any of the weekly check-in surveys was also associated with missing a care visit. While our study lacked the statistical power needed to assess whether disruptive life events reported less frequently, including housing insecurity, experiencing violence, and being arrested, were associated with poor outcomes, our results support the need to conduct larger studies of time-varying threats to care engagement in the future. An mHealth system like ours could be used in HIV care settings to assist case managers and social workers in proactively detecting and addressing these threats.

Our sample of people who use drugs answered an average of 23 of 52 total weekly surveys available to them during the 1-year follow-up period, a response rate of 44%. Despite that participants were not remunerated for completing weekly surveys, as they were operationalized as a self-assessment tool to enhance self-determined motivation as part of the A-CHESS app, this response rate is similar to several studies among people living with HIV [28]. Nonetheless, the utility of the weekly survey as an informative tool for clinical action may require a higher response rate. Thus, future studies may consider strategies such as incentivizing weekly surveys or sending surveys at a time most likely to get a response [29].

At the same time, our findings suggest that missing up to 4 consecutive surveys may be a helpful threshold to prompt a case manager or social worker to proactively reach out to a patient about any difficulties they may be experiencing and ensure any needs are met through appropriate referrals or direct provision of support. This conclusion is supported by considering multiple of our findings together, including our modest response rate, our finding that not answering at least 1 survey over a 4-week period was associated with missing a care visit, and our finding that those who did not experience viral nonsuppression or missed visits tended to engage with the app more frequently. A focus on monthly, rather than weekly, engagement with A-CHESS may also help to reduce alert fatigue, a phenomenon wherein people using technology become desensitized after receiving many alerts or notifications [30-33] and balance the extra staff time required to access A-CHESS and follow up with disengaged participants. Indeed, another ongoing study of A-CHESS in the substance use treatment setting suggests that staff time is a major barrier to using a clinician dashboard feature added to A-CHESS [34], which provides an easy-to-interpret interface to monitor patient survey results and app engagement. Prior studies of A-CHESS also suggest that providing dedicated support to implement A-CHESS within a health care organization using strategies such as coaching and process improvement models to ensure clinicians have dedicated time, technical assistance, and resources to integrate this new clinical tool into existing workflows [35,36]. Our finding that using and injecting drugs were associated with subsequently missing care visits further suggests that understanding the context of drug use in weekly surveys may assist with identifying supports available to app users at the time when drug use or craving occurs [37].

The need for novel and effective mHealth interventions is underscored by findings from several demonstration projects that were part of the Health Resources and Services Administration’s Special Projects of National Significance focused on social media and mHealth for supporting retention in HIV care [38]. This work identified 3 promising foci for mHealth tools, including (1) managing HIV care; (2) fostering feelings of support and personal connectedness; and (3) helping alleviate negative feelings about status and mitigating HIV-related stigma [39]. A-CHESS can meet all 3 of these needs. Through its theoretical foundations in self-determination theory [25], A-CHESS facilitates patient-provider connection and support through messaging functions and a clinician dashboard that can highlight weekly self-monitoring survey results. Peer connections are augmented by the use of a discussion board, targeted resources, and other key features.

### Limitations

The modest sample size of the study limits its internal and external validity and our ability to adjust for a broad set of confounders. Thus, we presented both unadjusted and adjusted results that could account for key confounders we identified a priori, including sociodemographic characteristics, comfort level with using a smartphone, and recent drug use at baseline. The overall conclusions drawn from the results were consistent in adjusted and unadjusted models. Additionally, several of the disruptive life events of interest (ie, housing instability, food insecurity, and law enforcement interaction) were reported infrequently over the follow-up period, which did not allow us to examine their association with missing a care visit or viral nonsuppression. As these are known contributors to disengagement with HIV care, they will be important to study in future, larger studies. Our results suggest that, when experienced, our smartphone app platform was able to capture at least some of these events on a weekly basis. Future research should also use qualitative methods to assess user experience, acceptability, and desired supports when disruptive life events occur. Finally, we were unable to include canceled care visits, and it was impossible to determine if or when the visit was rescheduled. Further analysis of patients who cancel a visit and do not return is warranted.

## Conclusions

A-CHESS is a promising tool for capturing time-varying threats to engagement in HIV care among people who use drugs. Future research should examine whether receiving tailored support based on disruptive life events reported in A-CHESS is effective in reducing viral nonsuppression and HIV care disengagement and evaluate how to integrate A-CHESS within the HIV medical home setting.

## Supplementary material

10.2196/59953 Multimedia Appendix 1 Supplemental tables.

10.2196/59953 Checklist 1 CONSORT (Consolidated Standards of Reporting Trials) checklist.

## Abbreviations

A-CHESS: Addiction Comprehensive Health Enhancement Support System
ALIVE: AIDS Linked to the Intravenous Experience
aOR: adjusted odds ratio
ART: antiretroviral therapy
CONSORT: Consolidated Standards of Reporting Trials
mHealth: mobile health
OUD: opioid use disorder
