# Supplementary material for: Mobile Health Tool to Capture Social Determinants of Health and Their Impact on HIV Treatment Outcomes Among People Who Use Drugs: Pilot Feasibility Study
Source: JMIR Form Res. 2025 Mar 26;9:e59953. doi: 10.2196/59953 (PMC11964955; doi:10.2196/59953)
Supplement: Multimedia Appendix 1 [file formative-v9-e59953-s001.docx]

**Supplemental Table 1.** Differences in A-CHESS utilization and weekly survey completion among those with and without virologic non-suppression and missed care visits in a study of 59 participants living with HIV and OUD enrolled in a pilot feasibility study of the A-CHESS mobile health application during 2017-2018

|  | Total | HIV Viral Suppression | | | Missed Care Visits | | |
| --- | --- | --- | --- | --- | --- | --- | --- |
|  | *N (%)* | *≥1 Test ≥200 copies/mL,*  *N (%)* | *All Tests <200 copies/mL,*  *N (%)* | *p-value* | *Missed ≥50% Visits,*  *N (%)* | *Missed <50% Visits,*  *N (%)* | *p-value* |
| Number of Participants | 59 | 25 | 27 | - | 23 | 33 | - |
| Times A-CHESS system used, Mean (SD)^a^ | 119.4 (82.8) | 111.8 (74.2) | 149.9 (83.3) | 0.088 | 94.4 (73.3) | 143.0 (84.2) | 0.026 |
| Times A-CHESS system used per month, Mean (SD) | 11.5 (4.4) | 10.9 (4.3) | 12.5 (4.6) | 0.18 | 10.2 (3.9) | 12.6 (4.7) | 0.040 |
| A-CHESS weekly surveys completed, Mean (SD) | 23.3 (16.3) | 19.7 (14.1) | 30.7 (15.9) | 0.011 | 16.8 (14.0) | 28.7 (16.3) | 0.0050 |
| A-CHESS weekly surveys per month, Mean (SD) | 2.68 (0.9) | 2.4 (0.9) | 3.0 (0.8) | 0.015 | 2.21 (0.8) | 3.04 (0.8) | <0.001 |

**Supplemental Table 2.** Association of reporting missed ART doses, drug use, and disruptive life events in the prior 30 days with HIV viral non-suppression (viral load ≥1,000 copies/mL) among 59 participants living with HIV and OUD enrolled in a pilot feasibility study of the A-CHESS mobile health application during 2017-2018

| Events reported in the month prior to viral load measurement^a^ | Unadjusted OR (95% CI) | Adjusted OR (95% CI)^b^ |
| --- | --- | --- |
| Any missed ART, drug use, or disruptive life event | 1.33 (0.59-3.03) | 1.41 (0.63-3.13) |
| 1 or more missed ART doses | 2.27 (0.91-5.88) | 2.22 (0.87-5.56) |
| 2 or more missed ART doses | 5.26 (1.96-14.29) | 4.76 (1.75-12.5) |
| Any drug use | 1.10 (0.44-2.70) | 1.20 (0.49-2.94) |
| Injected drugs | 0.59 (0.11-3.13) | 0.60 (0.10-3.57) |
| Did not have a place to sleep at night | 1.45 (0.05-50.0) | 1.49 (0.03-100.0) |
| Skipped a meal due to not enough money | 1.18 (0.30-4.76) | 1.18 (0.26-5.26) |
| Stopped by police | 1.06 (0.16-7.14) | 1.05 (0.17-6.67) |
| No weekly surveys completed in past 30 days | 1.15 (0.34-3.85) | 1.06 (0.30-3.85) |

^a^Odds ratios are reported relative to a referent group that completed at least one weekly survey in the 30 days prior to a scheduled HIV care visit but did not indicate experiencing the disruptive life event.

^b^Adjusted for age, gender, comfort using a smartphone (score: 1 [not at all comfortable] to 5 [very comfortable]), and any drug use in the past 30 days, all of which were reported at study enrollment.
